# Elementary Microscopic Technology

**Published:** 1887-09

**Authors:** 


ELEMENTARY MICROSCOPICAL TECHNOLOGY, a manual for students in microscopy; in three parts: Part I. The Technical History of a Slide, from the crude materials to the finished mount, by Frank L. James, Ph. D. M. D., President St. Louis Society Microscopists, Member American Society Microscopists, Editor St. Louis *Medical and Surgical Journal*, etc. St. Louis, Mo., published by the St. Louis Medical and Surgical Journal Co: 1887. Octavo, 106 pages, flexible covers.

Here is the very book that beginners in microscopy want; and now that the microscope has become an indispensable element in medicine, all should be beginners who are not now microscopists. The trouble with most text books is, that the authors forget that many readers know nothing about the subject; they suppose too much knowledge on the part of the reader. This trouble is obviated here. Dr. James evidently "knew how it was himself," and *begun* at the *beginning*; begun with the A B C of microscopy, as it were. Without a thorough knowledge of the "elementary technology," of microscopy, one might study the subject ever so diligently, and make but little headway. This is a useful book, and notwithstanding its very elementary nature, a microscopist may learn something from it.

J. WALTER THOMPSON'S LIST OF PROMINENT MEDICAL JOURNALS for the use of advertisers, is something new ; it is unique. The Journals selected for the list have each a *fac-simile* of its title page illustrated in the catalogue. It gives the advertiser a pretty good idea of the character of each publication. Thompson is evidently a live man, and deserves—and doubtless will achieve success in his line of business. Send for one of his pretty catalogues.

THE NEW ORLEANS POLYCLINIC.—We have received the first annual anouncement of the New Orleans Polyclinic, with the following corps of distinguished physicians as instructors : Drs. Rudolph Matas, F. W. Parham. J. H. Bemiss, G. B. Lawrason, P. E. Archinard, A. McShane, H. W. Blanc, H. D. Bruns, W. C. Ayres and Mr. J. Johnson. This is a good move, and we have wondered, why it has not been made before. The Charity Hospital furnishes an abundance of clinical material of all kinds, and our Southern teachers are second to none in native ability, even if less known than some in the north. Success to the Polyclinic.

